# Crystal structures of 4-chloro­phenyl *N*-(3,5-di­nitro­phen­yl)carbamate and phenyl *N*-(3,5-di­nitro­phen­yl)carbamate

**DOI:** 10.1107/S2056989015010245

**Published:** 2015-06-03

**Authors:** Rajamani Raja, Subramaniyan Sathiyaraj, B. Mohamad Ali, A. Sultan Nasar

**Affiliations:** a Department of Physics, Presidency College (Autonomous), Chennai 600 005, India; b Department of Polymer Science, University of Madras, Guindy campus, Chennai 602 025, India

**Keywords:** crystal structure, carbamate, 3,5-di­nitro­phenyl­carbamate, hydrogen bonding

## Abstract

The title compounds, C_13_H_8_ClN_3_O_6_, (I), and C_13_H_9_N_3_O_6_, (II), differ in the orientation of the two aromatic rings. In (I), they are essentially coplanar, making a dihedral angle of 8.2 (1)°, while in (II), they are inclined to one another by 76.2 (1)°. The two nitro groups are essentially coplanar with the attached benzene rings, as indicated by the dihedral angles of 1.4 (2) and 2.3 (2)° in (I), and 4.96 (18) and 5.4 (2)° in (II). The carbamate group is twisted slightly from the attached benzene ring, with a C—N—C—O torsion angle of −170.17 (15)° for (I) and 168.91 (13)° for (II). In the crystals of of both compounds, mol­ecules are linked *via* N—H⋯O hydrogen bonds, forming chains propagating along [010]. In (I), C—H⋯O hydrogen bonds also link mol­ecules within the chains. The crystal packing in (I) also features a very weak π–π inter­action [centroid–centroid distance = 3.7519 (9) Å].

## Abstract 2

In the title compounds, the planes of the two aromatic rings are inclined to one another by 7.60 (8) and76.19 (8)°. In the crystals of both compounds, mol­ecules are linked *via* N—H⋯O hydrogen bonds, forming chains along [010].

## Chemical context   

Carbamates are widely employed as pharmacological and therapeutic agents (Greig *et al.*, 2005 ▸) to inhibit different enzymes, such as acetyl- and butyrylcholinesterases (Darvesh *et al.*, 2008 ▸), cholesterol esterase (Hosie *et al.*, 1987 ▸), elastase (Digenis *et al.*, 1986 ▸,) chymotrypsin (Lin *et al.*, 2006 ▸) and fatty acid amide hydro­lase (FAAH) (Kathuria *et al.*, 2003 ▸). The therapeutic exploitation of the endocannabinoid system with exogenous agonists is limited by the undesired side effects caused by indiscriminate activation of cannabinoid type-1 (CB1) receptors, particularly in the brain (Mechoulam & Parker, 2013 ▸). An alternative strategy to direct CB1 receptor targeting is to increase the signaling activity of the endogenous cannabinoid ligands, arachidonoyl­ethano­lamide (anandamide) (Di Marzo *et al.*, 1994 ▸) and 2-arachidonoyl-sn-glycerol (2-AG) (Stella *et al.*, 1997 ▸), by blocking their intra­cellular degradation. As part of our studies in this area, we report herein on the syntheses and crystal structures of two 3,5-di­nitro­phenyl­carbamate derivatives, (I) and (II).
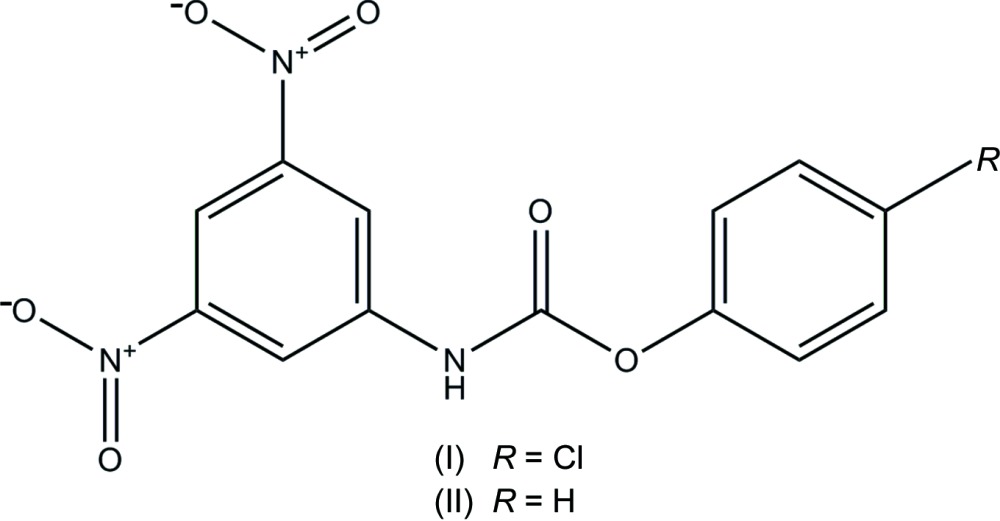


## Structural commentary   

**Figure 1:**
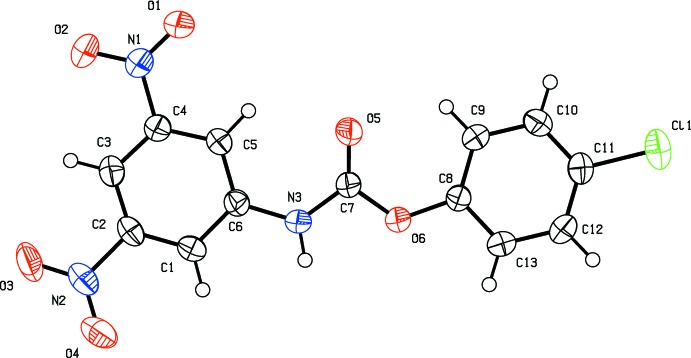
The mol­ecular structure of compound (I), showing the atom labelling. Displacement ellipsoids are drawn at the 50% probability level.

The mol­ecular structures of the title compounds, (I) and (II), are shown in Figs. 1 ▸ and 2 ▸, respectively. The molecules have different conformations. In compound (I), the benzene rings (C1–C6 and C8–C13) are almost coplanar, making a dihedral angle of 7.60 (8)°. The mean plane of the carbamate group (N3/C7/O5/O6) is twisted out of the planes of the rings by 14.00 (9) and 20.96 (9)°, respectively. In compound (II), the benzene and phenyl rings (C1–C6 and C8–C13, respectively) are roughly normal to one another, making a dihedral angle of 76.19 (8)°. Here, the mean plane of the carbamate group (N3/C7/O5/O6) is twisted out of the planes of the rings by 37.51 (8) and 80.90 (9)°, respectively.

## Supra­molecular features   

**Figure 3:**
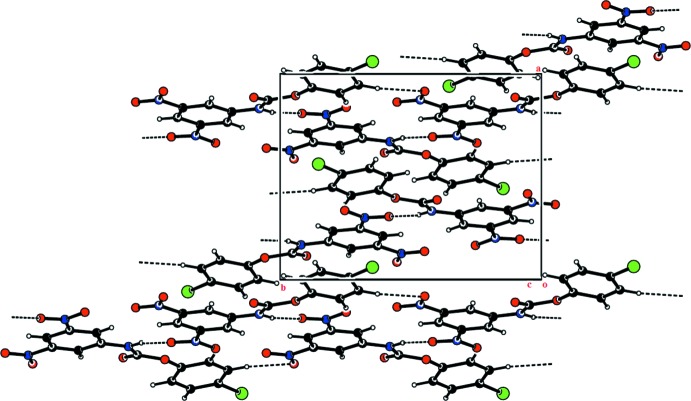
The crystal packing of compound (I), viewed along the *c* axis. The hydrogen bonds are shown as dashed lines (see Table 1 ▸ for details).

In the crystal of (I), N—H⋯O hydrogen bonds, involving a nitro O atom, O3, link adjacent mol­ecules into zigzag chains along the *b* axis (Table 1 ▸ and Fig. 3 ▸). Within the chain mol­ecules are also linked by C—H⋯O hydrogen bonds. The packing also features a very weak π–π inter­action [*Cg*1⋯*Cg*2^i^ = 3.7519 (9) Å; *Cg*1 and *Cg*2 are the centroids of rings C1–C6 and C8–C13, respectively; symmetry code: (i) −*x* + 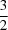, *y* + 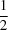, −*z* + 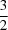].

**Figure 4:**
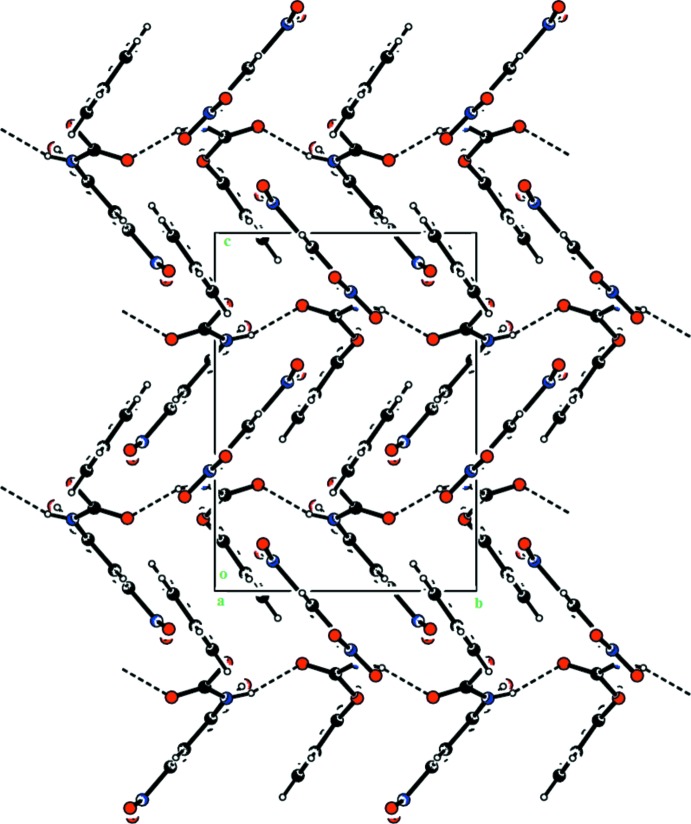
A view along the *a* axis of the crystal packing of compound (II). The hydrogen bonds are shown as dashed lines (see Table 2 ▸ for details).

In the crystal of (II), mol­ecules are again linked *via* N—H⋯O hydrogen bonds, this time involving the carbonyl O atom O5, forming chains propagating along the *b* axis; see Table 2 ▸ and Fig. 4 ▸.

## Database survey   

A search of the Cambridge Structural Database (Version 5.36, February 2015; Groom & Allen, 2014 ▸) for phenyl *N*-phenyl­carbamate gave 16 hits for similar compounds, including two ortho­rhom­bic poylmorphs of phenyl *N*-phenyl­carbamate itself (YEHPOQ: Lehr *et al.*, 2001 ▸; YEHPOQ01; Shahwar *et al.*, 2009 ▸). In the first polymorph (YEHPOQ), the phenyl rings are inclined to one another by 25.76°, while in the latter (YEHPOQ01) the equivalent dihedral angle is 42.50°. These values are quite different to those observed for compounds (I) and (II); *cf.* 7.60 (8)° in (I), and 76.19 (8)° in (II).

## Synthesis and crystallization   

The title compounds were prepared in a similar manner using a stirred solution of of 3,5 di­nitro­aniline (1.0 g, 5.45 mmol) dissolved in 100 ml of dry THF, and to it was added the calculated amount (with 5% excess) of 4-chloro­phenyl­chloro­formate for compound (I), or phenyl­chloro­formate for compound (II), dissolved in 50 ml of dry THF. The addition rate was such that it took 90 min for complete transfer of 4-chlorophenylchloroformate for compound (I), and phenylchloroformate for compound (II). After the addition was over, stirring was continued overnight. Excess THF was removed under vacuum at room temperature. The crude product was extracted with ethyl acetate (3 × 100 ml). The organic layer was dried over anhydrous sodium sulfate. Removal of solvent under vacuum at room temperature yielded a light-yellow product. It was dried under vacuum to constant weight. It was dissolved in ethyl acetate and just warmed-up using a water bath, and then kept at room temperature. The solvent was slowly evaporated and light-yellow crystals of each of the title compounds were obtained (yields 99%).

## Refinement details   

Crystal data, data collection and structure refinement details are summarized in Table 3 ▸. The N- and C-bound H atoms were positioned geometrically (N—H = 0.86 Å, C—H = 0.93 Å) and allowed to ride on their parent atoms, with *U*_iso_(H) = 1.2*U*_eq_(N,C).

## Supplementary Material

Crystal structure: contains datablock(s) global, I, II. DOI: 10.1107/S2056989015010245/su5141sup1.cif

Structure factors: contains datablock(s) I. DOI: 10.1107/S2056989015010245/su5141Isup2.hkl

Structure factors: contains datablock(s) II. DOI: 10.1107/S2056989015010245/su5141IIsup3.hkl

Supporting information file. DOI: 10.1107/S2056989015010245/su5141Isup4.cml

Supporting information file. DOI: 10.1107/S2056989015010245/su5141IIsup5.cml

CCDC references: 1403525, 1403524

Additional supporting information:  crystallographic information; 3D view; checkCIF report

## Figures and Tables

**Figure 2:**
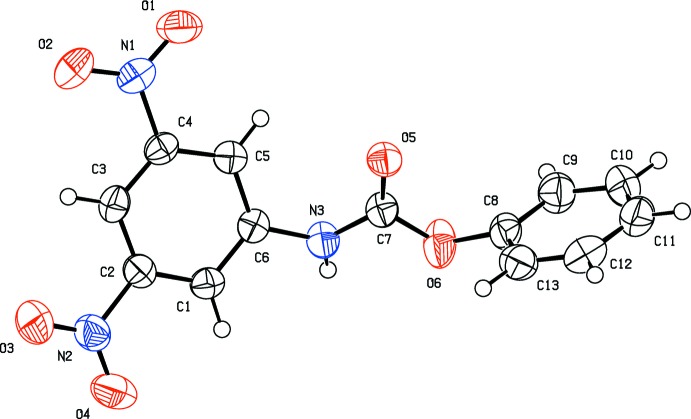
The mol­ecular structure of compound (II), showing the atom labelling. Displacement ellipsoids are drawn at the 50% probability level.

**Table 1:** Hydrogen-bond geometry (Å, °) for (I)

*D*—H⋯*A*	*D*—H	H⋯*A*	*D*⋯*A*	*D*—H⋯*A*
N3—H3*A*⋯O3^i^	0.86	2.18	3.0286 (19)	168
C12—H12⋯O1^ii^	0.93	2.54	3.428 (2)	159

Symmetry codes: (i) 
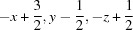
; (ii) 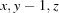.

**Table 2:** Hydrogen-bond geometry (Å, °) for (II)

*D*—H⋯*A*	*D*—H	H⋯*A*	*D*⋯*A*	*D*—H⋯*A*
N3—H3*A*⋯O5^i^	0.86	2.07	2.8836 (15)	157

Symmetry code: (i) 
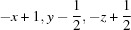
.

**Table 3:** Experimental details

	(I)	(II)
Crystal data		
Chemical formula	C_13_H_8_ClN_3_O_6_	C_13_H_9_N_3_O_6_
*M*_r_	337.67	303.23
Crystal system, space group	Monoclinic, *P*2_1_/*n*	Monoclinic, *P*2_1_/*c*
Temperature (K)	293	293
*a*, *b*, *c* (Å)	9.9103 (4), 12.5791 (4), 10.9772 (5)	12.2549 (4), 8.8717 (4), 12.1470 (5)
β (°)	94.183 (2)	91.673 (2)
*V* (Å^3^)	1364.80 (9)	1320.08 (9)
*Z*	4	4
Radiation type	Mo *K*α	Mo *K*α
μ (mm^−1^)	0.32	0.12
Crystal size (mm)	0.35 × 0.30 × 0.25	0.35 × 0.30 × 0.25

Data collection		
Diffractometer	Bruker SMART APEXII CCD	Bruker SMART APEXII CCD
Absorption correction	Multi-scan (*SADABS*; Bruker, 2008 ▸)	Multi-scan (*SADABS*; Bruker, 2008 ▸)
*T*_min_, *T*_max_	0.938, 0.944	0.969, 0.976
No. of measured, independent and observed [*I* > 2σ(*I*)] reflections	8697, 2584, 2134	11395, 2925, 2355
*R*_int_	0.015	0.020
(sin θ/λ)_max_ (Å^−1^)	0.610	0.642

Refinement		
*R*[*F* ^2^ > 2σ(*F* ^2^)], *wR*(*F* ^2^), *S*	0.033, 0.086, 1.04	0.044, 0.122, 1.03
No. of reflections	2584	2925
No. of parameters	208	199
H-atom treatment	H-atom parameters constrained	H-atom parameters constrained
Δρ_max_, Δρ_min_ (e Å^−3^)	0.18, −0.20	0.23, −0.27

Computer programs: *APEX2* and *SAINT* (Bruker, 2008 ▸), *SHELXS97* (Sheldrick, 2008 ▸), *SHELXL2014* (Sheldrick, 2015 ▸) and *PLATON* (Spek, 2009 ▸).

## supplementary crystallographic information

### (I) 4-Chlorophenyl N-(3,5-dinitrophenyl)carbamate Crystal data

**Table:**

C_13_H_8_ClN_3_O_6_	*F*(000) = 688
*M**_r_* = 337.67	*D*_x_ = 1.643 Mg m^−^^3^
Monoclinic, *P*2_1_/*n*	Mo *K*α radiation, λ = 0.71073 Å
*a* = 9.9103 (4) Å	Cell parameters from 2013 reflections
*b* = 12.5791 (4) Å	θ = 2.5–25.0°
*c* = 10.9772 (5) Å	µ = 0.32 mm^−^^1^
β = 94.183 (2)°	*T* = 293 K
*V* = 1364.80 (9) Å^3^	Block, yellow
*Z* = 4	0.35 × 0.30 × 0.25 mm

### (I) 4-Chlorophenyl N-(3,5-dinitrophenyl)carbamate Data collection

**Table:**

Bruker SMART APEXII CCD diffractometer	2584 independent reflections
Radiation source: fine-focus sealed tube	2134 reflections with *I* > 2σ(*I*)
Graphite monochromator	*R*_int_ = 0.015
ω and φ scans	θ_max_ = 25.7°, θ_min_ = 2.5°
Absorption correction: multi-scan (*SADABS*; Bruker, 2008)	*h* = −12→12
*T*_min_ = 0.938, *T*_max_ = 0.944	*k* = −15→9
8697 measured reflections	*l* = −13→11

### (I) 4-Chlorophenyl N-(3,5-dinitrophenyl)carbamate Refinement

**Table:**

Refinement on *F*^2^	Primary atom site location: structure-invariant direct methods
Least-squares matrix: full	Secondary atom site location: difference Fourier map
*R*[*F*^2^ > 2σ(*F*^2^)] = 0.033	Hydrogen site location: inferred from neighbouring sites
*wR*(*F*^2^) = 0.086	H-atom parameters constrained
*S* = 1.04	*w* = 1/[σ^2^(*F*_o_^2^) + (0.0379*P*)^2^ + 0.4574*P*] where *P* = (*F*_o_^2^ + 2*F*_c_^2^)/3
2584 reflections	(Δ/σ)_max_ = 0.001
208 parameters	Δρ_max_ = 0.18 e Å^−^^3^
0 restraints	Δρ_min_ = −0.20 e Å^−^^3^

### (I) 4-Chlorophenyl N-(3,5-dinitrophenyl)carbamate Special details

**Table:**

Geometry. All esds (except the esd in the dihedral angle between two l.s. planes) are estimated using the full covariance matrix. The cell esds are taken into account individually in the estimation of esds in distances, angles and torsion angles; correlations between esds in cell parameters are only used when they are defined by crystal symmetry. An approximate (isotropic) treatment of cell esds is used for estimating esds involving l.s. planes.

### (I) 4-Chlorophenyl N-(3,5-dinitrophenyl)carbamate Fractional atomic coordinates and isotropic or equivalent isotropic displacement parameters (Å^2^)

**Table:**

	*x*	*y*	*z*	*U*_iso_*/*U*_eq_	
Cl1	1.06146 (5)	−0.35304 (4)	1.04085 (5)	0.06328 (18)	
O1	0.9159 (2)	0.44697 (12)	0.70274 (14)	0.0966 (7)	
O2	0.86323 (17)	0.55251 (10)	0.55446 (13)	0.0687 (4)	
O3	0.69275 (17)	0.41624 (12)	0.16853 (12)	0.0701 (4)	
O4	0.66305 (14)	0.24728 (12)	0.14188 (12)	0.0629 (4)	
O5	0.88633 (15)	0.10055 (9)	0.73898 (11)	0.0570 (4)	
O6	0.89504 (13)	−0.05893 (9)	0.64269 (10)	0.0471 (3)	
N1	0.87388 (17)	0.46386 (12)	0.59848 (14)	0.0514 (4)	
N2	0.69860 (15)	0.32352 (13)	0.20431 (13)	0.0472 (4)	
N3	0.83153 (15)	0.07996 (10)	0.53417 (12)	0.0421 (3)	
H3A	0.8133	0.0312	0.4806	0.051*	
C1	0.76419 (15)	0.20217 (13)	0.37383 (14)	0.0367 (4)	
H1	0.7386	0.1452	0.3234	0.044*	
C2	0.75199 (15)	0.30533 (13)	0.33135 (14)	0.0369 (4)	
C3	0.78764 (16)	0.39277 (13)	0.40121 (15)	0.0403 (4)	
H3	0.7800	0.4615	0.3704	0.048*	
C4	0.83533 (16)	0.37228 (12)	0.51973 (14)	0.0376 (4)	
C5	0.85006 (16)	0.27193 (12)	0.56865 (14)	0.0367 (4)	
H5	0.8822	0.2623	0.6496	0.044*	
C6	0.81578 (15)	0.18518 (12)	0.49431 (14)	0.0344 (3)	
C7	0.87274 (16)	0.04673 (12)	0.64903 (14)	0.0362 (4)	
C8	0.93362 (16)	−0.12190 (12)	0.74459 (14)	0.0347 (4)	
C9	1.00067 (18)	−0.08512 (13)	0.85107 (16)	0.0441 (4)	
H9	1.0194	−0.0131	0.8613	0.053*	
C10	1.03967 (18)	−0.15731 (14)	0.94259 (16)	0.0454 (4)	
H10	1.0840	−0.1339	1.0153	0.054*	
C11	1.01246 (16)	−0.26366 (13)	0.92519 (15)	0.0400 (4)	
C12	0.94754 (17)	−0.30018 (13)	0.81784 (16)	0.0420 (4)	
H12	0.9309	−0.3724	0.8067	0.050*	
C13	0.90742 (16)	−0.22847 (12)	0.72698 (15)	0.0396 (4)	
H13	0.8630	−0.2520	0.6544	0.048*	

### (I) 4-Chlorophenyl N-(3,5-dinitrophenyl)carbamate Atomic displacement parameters (Å^2^)

**Table:**

	*U*^11^	*U*^22^	*U*^33^	*U*^12^	*U*^13^	*U*^23^
Cl1	0.0702 (3)	0.0569 (3)	0.0615 (3)	0.0118 (2)	−0.0038 (2)	0.0230 (2)
O1	0.192 (2)	0.0443 (8)	0.0469 (9)	−0.0131 (10)	−0.0372 (11)	−0.0008 (7)
O2	0.1083 (12)	0.0319 (7)	0.0639 (9)	−0.0004 (7)	−0.0065 (8)	0.0048 (6)
O3	0.1021 (12)	0.0657 (9)	0.0412 (8)	0.0195 (8)	−0.0030 (7)	0.0199 (7)
O4	0.0672 (9)	0.0793 (10)	0.0400 (7)	0.0021 (7)	−0.0117 (6)	−0.0028 (7)
O5	0.0993 (11)	0.0377 (7)	0.0322 (7)	0.0145 (7)	−0.0061 (6)	−0.0037 (5)
O6	0.0763 (9)	0.0307 (6)	0.0328 (6)	0.0004 (5)	−0.0069 (6)	−0.0011 (5)
N1	0.0727 (11)	0.0354 (8)	0.0450 (9)	−0.0033 (7)	−0.0017 (8)	0.0009 (7)
N2	0.0461 (8)	0.0636 (10)	0.0318 (8)	0.0092 (7)	0.0012 (6)	0.0052 (7)
N3	0.0630 (9)	0.0325 (7)	0.0297 (7)	−0.0024 (6)	−0.0047 (6)	−0.0033 (6)
C1	0.0384 (8)	0.0430 (9)	0.0288 (8)	−0.0005 (7)	0.0023 (6)	−0.0025 (7)
C2	0.0338 (8)	0.0497 (9)	0.0274 (8)	0.0056 (7)	0.0030 (6)	0.0057 (7)
C3	0.0440 (9)	0.0392 (9)	0.0378 (9)	0.0038 (7)	0.0042 (7)	0.0075 (7)
C4	0.0428 (9)	0.0352 (8)	0.0347 (9)	−0.0007 (7)	0.0025 (7)	−0.0001 (7)
C5	0.0420 (9)	0.0371 (8)	0.0305 (8)	−0.0006 (7)	−0.0012 (6)	0.0025 (7)
C6	0.0375 (8)	0.0348 (8)	0.0311 (8)	0.0004 (6)	0.0030 (6)	0.0019 (6)
C7	0.0464 (9)	0.0310 (8)	0.0310 (9)	0.0007 (7)	0.0011 (7)	0.0006 (7)
C8	0.0411 (9)	0.0305 (8)	0.0325 (8)	0.0012 (6)	0.0027 (7)	0.0006 (6)
C9	0.0571 (11)	0.0307 (8)	0.0430 (10)	−0.0022 (7)	−0.0068 (8)	−0.0027 (7)
C10	0.0516 (10)	0.0453 (10)	0.0375 (9)	0.0033 (8)	−0.0085 (8)	−0.0023 (8)
C11	0.0390 (9)	0.0389 (9)	0.0422 (9)	0.0054 (7)	0.0041 (7)	0.0070 (7)
C12	0.0467 (9)	0.0295 (8)	0.0498 (10)	−0.0020 (7)	0.0041 (8)	−0.0005 (7)
C13	0.0448 (9)	0.0341 (8)	0.0394 (9)	−0.0028 (7)	−0.0010 (7)	−0.0055 (7)

### (I) 4-Chlorophenyl N-(3,5-dinitrophenyl)carbamate Geometric parameters (Å, º)

**Table:**

Cl1—C11	1.7384 (16)	C2—C3	1.372 (2)
O1—N1	1.208 (2)	C3—C4	1.376 (2)
O2—N1	1.2169 (19)	C3—H3	0.9300
O3—N2	1.231 (2)	C4—C5	1.375 (2)
O4—N2	1.216 (2)	C5—C6	1.390 (2)
O5—C7	1.1967 (19)	C5—H5	0.9300
O6—C7	1.3500 (19)	C8—C13	1.376 (2)
O6—C8	1.4009 (19)	C8—C9	1.381 (2)
N1—C4	1.474 (2)	C9—C10	1.388 (2)
N2—C2	1.473 (2)	C9—H9	0.9300
N3—C7	1.362 (2)	C10—C11	1.375 (2)
N3—C6	1.399 (2)	C10—H10	0.9300
N3—H3A	0.8600	C11—C12	1.380 (2)
C1—C2	1.381 (2)	C12—C13	1.382 (2)
C1—C6	1.399 (2)	C12—H12	0.9300
C1—H1	0.9300	C13—H13	0.9300
			
C7—O6—C8	123.48 (12)	C5—C6—N3	122.83 (14)
O1—N1—O2	123.46 (16)	C5—C6—C1	119.46 (15)
O1—N1—C4	118.36 (14)	N3—C6—C1	117.71 (14)
O2—N1—C4	118.17 (15)	O5—C7—O6	126.20 (15)
O4—N2—O3	124.24 (15)	O5—C7—N3	126.73 (15)
O4—N2—C2	118.71 (15)	O6—C7—N3	107.07 (13)
O3—N2—C2	117.05 (16)	C13—C8—C9	121.28 (15)
C7—N3—C6	126.78 (13)	C13—C8—O6	113.63 (14)
C7—N3—H3A	116.6	C9—C8—O6	124.94 (14)
C6—N3—H3A	116.6	C8—C9—C10	118.99 (15)
C2—C1—C6	118.66 (15)	C8—C9—H9	120.5
C2—C1—H1	120.7	C10—C9—H9	120.5
C6—C1—H1	120.7	C11—C10—C9	119.63 (16)
C3—C2—C1	123.49 (15)	C11—C10—H10	120.2
C3—C2—N2	117.68 (15)	C9—C10—H10	120.2
C1—C2—N2	118.83 (15)	C10—C11—C12	121.14 (15)
C2—C3—C4	115.78 (15)	C10—C11—Cl1	119.09 (13)
C2—C3—H3	122.1	C12—C11—Cl1	119.77 (13)
C4—C3—H3	122.1	C11—C12—C13	119.40 (15)
C5—C4—C3	124.09 (15)	C11—C12—H12	120.3
C5—C4—N1	118.22 (14)	C13—C12—H12	120.3
C3—C4—N1	117.69 (14)	C8—C13—C12	119.55 (15)
C4—C5—C6	118.48 (14)	C8—C13—H13	120.2
C4—C5—H5	120.8	C12—C13—H13	120.2
C6—C5—H5	120.8		
			
C6—C1—C2—C3	−0.1 (2)	C7—N3—C6—C1	−176.15 (15)
C6—C1—C2—N2	179.58 (14)	C2—C1—C6—C5	1.6 (2)
O4—N2—C2—C3	−178.06 (15)	C2—C1—C6—N3	−177.90 (14)
O3—N2—C2—C3	1.7 (2)	C8—O6—C7—O5	2.4 (3)
O4—N2—C2—C1	2.2 (2)	C8—O6—C7—N3	−177.36 (14)
O3—N2—C2—C1	−177.98 (15)	C6—N3—C7—O5	10.1 (3)
C1—C2—C3—C4	−1.1 (2)	C6—N3—C7—O6	−170.17 (15)
N2—C2—C3—C4	179.18 (14)	C7—O6—C8—C13	159.36 (15)
C2—C3—C4—C5	1.0 (2)	C7—O6—C8—C9	−25.2 (2)
C2—C3—C4—N1	−179.54 (14)	C13—C8—C9—C10	−1.3 (3)
O1—N1—C4—C5	−0.5 (3)	O6—C8—C9—C10	−176.42 (15)
O2—N1—C4—C5	178.63 (16)	C8—C9—C10—C11	0.8 (3)
O1—N1—C4—C3	179.99 (19)	C9—C10—C11—C12	0.4 (3)
O2—N1—C4—C3	−0.9 (2)	C9—C10—C11—Cl1	−179.80 (14)
C3—C4—C5—C6	0.4 (2)	C10—C11—C12—C13	−1.0 (3)
N1—C4—C5—C6	−179.06 (14)	Cl1—C11—C12—C13	179.19 (13)
C4—C5—C6—N3	177.72 (15)	C9—C8—C13—C12	0.7 (2)
C4—C5—C6—C1	−1.7 (2)	O6—C8—C13—C12	176.33 (15)
C7—N3—C6—C5	4.4 (3)	C11—C12—C13—C8	0.4 (2)

### (I) 4-Chlorophenyl N-(3,5-dinitrophenyl)carbamate Hydrogen-bond geometry (Å, º)

**Table:**

*D*—H···*A*	*D*—H	H···*A*	*D*···*A*	*D*—H···*A*
N3—H3*A*···O3^i^	0.86	2.18	3.0286 (19)	168
C12—H12···O1^ii^	0.93	2.54	3.428 (2)	159

Symmetry codes: (i) −*x*+3/2, *y*−1/2, −*z*+1/2; (ii) *x*, *y*−1, *z*.

### (II) Phenyl N-(3,5-dinitrophenyl)carbamate Crystal data

**Table:**

C_13_H_9_N_3_O_6_	*F*(000) = 624
*M**_r_* = 303.23	*D*_x_ = 1.526 Mg m^−^^3^
Monoclinic, *P*2_1_/*c*	Mo *K*α radiation, λ = 0.71073 Å
*a* = 12.2549 (4) Å	Cell parameters from 1992 reflections
*b* = 8.8717 (4) Å	θ = 1.7–25.0°
*c* = 12.1470 (5) Å	µ = 0.12 mm^−^^1^
β = 91.673 (2)°	*T* = 293 K
*V* = 1320.08 (9) Å^3^	Block, yellow
*Z* = 4	0.35 × 0.30 × 0.25 mm

### (II) Phenyl N-(3,5-dinitrophenyl)carbamate Data collection

**Table:**

Bruker SMART APEXII CCD diffractometer	2925 independent reflections
Radiation source: fine-focus sealed tube	2355 reflections with *I* > 2σ(*I*)
Graphite monochromator	*R*_int_ = 0.020
ω and φ scans	θ_max_ = 27.1°, θ_min_ = 1.7°
Absorption correction: multi-scan (*SADABS*; Bruker, 2008)	*h* = −13→15
*T*_min_ = 0.969, *T*_max_ = 0.976	*k* = −7→11
11395 measured reflections	*l* = −15→15

### (II) Phenyl N-(3,5-dinitrophenyl)carbamate Refinement

**Table:**

Refinement on *F*^2^	Primary atom site location: structure-invariant direct methods
Least-squares matrix: full	Secondary atom site location: difference Fourier map
*R*[*F*^2^ > 2σ(*F*^2^)] = 0.044	Hydrogen site location: inferred from neighbouring sites
*wR*(*F*^2^) = 0.122	H-atom parameters constrained
*S* = 1.03	*w* = 1/[σ^2^(*F*_o_^2^) + (0.0617*P*)^2^ + 0.328*P*] where *P* = (*F*_o_^2^ + 2*F*_c_^2^)/3
2925 reflections	(Δ/σ)_max_ < 0.001
199 parameters	Δρ_max_ = 0.23 e Å^−^^3^
0 restraints	Δρ_min_ = −0.27 e Å^−^^3^

### (II) Phenyl N-(3,5-dinitrophenyl)carbamate Special details

**Table:**

Geometry. All esds (except the esd in the dihedral angle between two l.s. planes) are estimated using the full covariance matrix. The cell esds are taken into account individually in the estimation of esds in distances, angles and torsion angles; correlations between esds in cell parameters are only used when they are defined by crystal symmetry. An approximate (isotropic) treatment of cell esds is used for estimating esds involving l.s. planes.

### (II) Phenyl N-(3,5-dinitrophenyl)carbamate Fractional atomic coordinates and isotropic or equivalent isotropic displacement parameters (Å^2^)

**Table:**

	*x*	*y*	*z*	*U*_iso_*/*U*_eq_	
O1	0.61513 (10)	0.32392 (15)	0.60653 (10)	0.0637 (4)	
O2	0.79022 (12)	0.31538 (18)	0.63113 (11)	0.0785 (4)	
O3	1.00811 (10)	0.0409 (2)	0.37541 (12)	0.0824 (5)	
O4	0.93014 (11)	−0.11517 (17)	0.26319 (12)	0.0722 (4)	
O5	0.42568 (8)	0.16533 (11)	0.29697 (8)	0.0433 (3)	
O6	0.38141 (10)	−0.04444 (13)	0.20033 (12)	0.0667 (4)	
N1	0.70568 (11)	0.27772 (15)	0.58344 (10)	0.0488 (3)	
N2	0.92743 (10)	−0.01973 (18)	0.33498 (11)	0.0522 (4)	
N3	0.53349 (9)	−0.04417 (13)	0.30003 (10)	0.0416 (3)	
H3A	0.5327	−0.1390	0.2852	0.050*	
C1	0.72796 (11)	−0.03251 (16)	0.32278 (11)	0.0382 (3)	
H5	0.7340	−0.1028	0.2665	0.046*	
C2	0.81974 (11)	0.02594 (17)	0.37459 (11)	0.0397 (3)	
C3	0.81594 (12)	0.12779 (17)	0.46001 (11)	0.0423 (3)	
H3	0.8789	0.1657	0.4944	0.051*	
C4	0.71342 (12)	0.17011 (15)	0.49145 (11)	0.0382 (3)	
C5	0.61827 (11)	0.11716 (15)	0.44186 (11)	0.0370 (3)	
H1	0.5506	0.1491	0.4655	0.044*	
C6	0.62602 (11)	0.01493 (14)	0.35580 (11)	0.0352 (3)	
C7	0.44606 (11)	0.03875 (15)	0.26836 (12)	0.0383 (3)	
C8	0.29939 (12)	0.03219 (17)	0.13844 (14)	0.0475 (4)	
C9	0.19265 (13)	0.0095 (2)	0.16418 (15)	0.0569 (4)	
H13	0.1747	−0.0481	0.2250	0.068*	
C10	0.11244 (14)	0.0740 (2)	0.09776 (16)	0.0644 (5)	
H12	0.0394	0.0593	0.1137	0.077*	
C11	0.13885 (15)	0.1595 (2)	0.00871 (15)	0.0610 (5)	
H11	0.0839	0.2024	−0.0354	0.073*	
C12	0.24650 (15)	0.1821 (2)	−0.01578 (14)	0.0590 (4)	
H10	0.2643	0.2407	−0.0761	0.071*	
C13	0.32810 (14)	0.1174 (2)	0.04939 (15)	0.0548 (4)	
H9	0.4011	0.1314	0.0333	0.066*	

### (II) Phenyl N-(3,5-dinitrophenyl)carbamate Atomic displacement parameters (Å^2^)

**Table:**

	*U*^11^	*U*^22^	*U*^33^	*U*^12^	*U*^13^	*U*^23^
O1	0.0677 (8)	0.0644 (8)	0.0598 (7)	0.0019 (6)	0.0147 (6)	−0.0192 (6)
O2	0.0757 (9)	0.0913 (11)	0.0673 (8)	−0.0013 (8)	−0.0177 (7)	−0.0362 (8)
O3	0.0406 (6)	0.1277 (14)	0.0791 (9)	−0.0059 (7)	0.0041 (6)	−0.0198 (9)
O4	0.0600 (8)	0.0823 (10)	0.0753 (8)	0.0092 (7)	0.0192 (6)	−0.0170 (8)
O5	0.0452 (6)	0.0320 (5)	0.0525 (6)	0.0027 (4)	−0.0015 (4)	−0.0037 (4)
O6	0.0591 (7)	0.0358 (6)	0.1029 (10)	0.0050 (5)	−0.0356 (7)	−0.0157 (6)
N1	0.0620 (8)	0.0448 (7)	0.0395 (6)	−0.0031 (6)	−0.0005 (6)	−0.0039 (6)
N2	0.0439 (7)	0.0672 (9)	0.0459 (7)	0.0044 (7)	0.0064 (6)	0.0044 (7)
N3	0.0427 (6)	0.0257 (6)	0.0559 (7)	−0.0010 (5)	−0.0061 (5)	−0.0023 (5)
C1	0.0466 (7)	0.0346 (7)	0.0334 (6)	0.0033 (6)	0.0020 (6)	0.0020 (5)
C2	0.0394 (7)	0.0435 (8)	0.0363 (7)	0.0038 (6)	0.0033 (5)	0.0081 (6)
C3	0.0428 (7)	0.0469 (8)	0.0369 (7)	−0.0040 (6)	−0.0053 (6)	0.0043 (6)
C4	0.0484 (7)	0.0344 (7)	0.0319 (6)	−0.0004 (6)	0.0000 (5)	0.0018 (5)
C5	0.0418 (7)	0.0318 (7)	0.0376 (7)	0.0009 (6)	0.0027 (5)	0.0044 (5)
C6	0.0411 (7)	0.0272 (6)	0.0371 (7)	−0.0011 (5)	−0.0019 (5)	0.0055 (5)
C7	0.0381 (7)	0.0293 (7)	0.0475 (7)	−0.0052 (5)	−0.0012 (6)	0.0007 (6)
C8	0.0434 (8)	0.0373 (8)	0.0610 (9)	0.0015 (6)	−0.0114 (7)	−0.0130 (7)
C9	0.0516 (9)	0.0643 (11)	0.0546 (9)	−0.0033 (8)	−0.0007 (7)	0.0016 (8)
C10	0.0403 (8)	0.0860 (14)	0.0667 (11)	0.0024 (8)	−0.0011 (8)	−0.0057 (10)
C11	0.0602 (10)	0.0674 (12)	0.0545 (10)	0.0101 (9)	−0.0143 (8)	−0.0082 (9)
C12	0.0751 (12)	0.0563 (10)	0.0457 (9)	−0.0051 (9)	0.0018 (8)	−0.0071 (8)
C13	0.0453 (8)	0.0522 (9)	0.0670 (10)	−0.0069 (7)	0.0061 (7)	−0.0168 (8)

### (II) Phenyl N-(3,5-dinitrophenyl)carbamate Geometric parameters (Å, º)

**Table:**

O1—N1	1.2232 (17)	C3—C4	1.376 (2)
O2—N1	1.2188 (17)	C3—H3	0.9300
O3—N2	1.2165 (19)	C4—C5	1.3791 (19)
O4—N2	1.2167 (19)	C5—C6	1.3894 (19)
O5—C7	1.2039 (16)	C5—H1	0.9300
O6—C7	1.3480 (17)	C8—C9	1.369 (2)
O6—C8	1.4119 (18)	C8—C13	1.374 (3)
N1—C4	1.4748 (18)	C9—C10	1.378 (2)
N2—C2	1.4745 (18)	C9—H13	0.9300
N3—C7	1.3465 (18)	C10—C11	1.368 (3)
N3—C6	1.4054 (17)	C10—H12	0.9300
N3—H3A	0.8600	C11—C12	1.376 (3)
C1—C2	1.374 (2)	C11—H11	0.9300
C1—C6	1.3885 (19)	C12—C13	1.382 (2)
C1—H5	0.9300	C12—H10	0.9300
C2—C3	1.378 (2)	C13—H9	0.9300
			
C7—O6—C8	117.34 (11)	C1—C6—C5	119.81 (12)
O2—N1—O1	124.29 (14)	C1—C6—N3	117.89 (12)
O2—N1—C4	117.71 (13)	C5—C6—N3	122.30 (12)
O1—N1—C4	118.00 (13)	O5—C7—N3	126.67 (13)
O3—N2—O4	123.94 (14)	O5—C7—O6	124.37 (13)
O3—N2—C2	118.12 (14)	N3—C7—O6	108.94 (12)
O4—N2—C2	117.94 (14)	C9—C8—C13	121.95 (15)
C7—N3—C6	123.92 (11)	C9—C8—O6	118.54 (16)
C7—N3—H3A	118.0	C13—C8—O6	119.31 (15)
C6—N3—H3A	118.0	C8—C9—C10	118.38 (17)
C2—C1—C6	118.98 (13)	C8—C9—H13	120.8
C2—C1—H5	120.5	C10—C9—H13	120.8
C6—C1—H5	120.5	C11—C10—C9	120.82 (16)
C1—C2—C3	123.17 (13)	C11—C10—H12	119.6
C1—C2—N2	118.37 (13)	C9—C10—H12	119.6
C3—C2—N2	118.44 (13)	C10—C11—C12	120.16 (17)
C4—C3—C2	116.06 (13)	C10—C11—H11	119.9
C4—C3—H3	122.0	C12—C11—H11	119.9
C2—C3—H3	122.0	C11—C12—C13	119.89 (17)
C3—C4—C5	123.57 (13)	C11—C12—H10	120.1
C3—C4—N1	117.80 (13)	C13—C12—H10	120.1
C5—C4—N1	118.63 (12)	C8—C13—C12	118.81 (16)
C4—C5—C6	118.39 (13)	C8—C13—H9	120.6
C4—C5—H1	120.8	C12—C13—H9	120.6
C6—C5—H1	120.8		
			
C6—C1—C2—C3	1.4 (2)	C4—C5—C6—C1	0.73 (19)
C6—C1—C2—N2	−177.01 (12)	C4—C5—C6—N3	−179.58 (12)
O3—N2—C2—C1	174.49 (15)	C7—N3—C6—C1	−137.09 (14)
O4—N2—C2—C1	−5.0 (2)	C7—N3—C6—C5	43.2 (2)
O3—N2—C2—C3	−4.0 (2)	C6—N3—C7—O5	−12.7 (2)
O4—N2—C2—C3	176.50 (14)	C6—N3—C7—O6	168.91 (13)
C1—C2—C3—C4	−0.4 (2)	C8—O6—C7—O5	15.9 (2)
N2—C2—C3—C4	178.04 (12)	C8—O6—C7—N3	−165.73 (14)
C2—C3—C4—C5	−0.5 (2)	C7—O6—C8—C9	−110.20 (18)
C2—C3—C4—N1	179.34 (12)	C7—O6—C8—C13	74.81 (19)
O2—N1—C4—C3	−4.8 (2)	C13—C8—C9—C10	0.4 (3)
O1—N1—C4—C3	174.88 (14)	O6—C8—C9—C10	−174.48 (15)
O2—N1—C4—C5	175.11 (14)	C8—C9—C10—C11	−0.4 (3)
O1—N1—C4—C5	−5.3 (2)	C9—C10—C11—C12	0.0 (3)
C3—C4—C5—C6	0.3 (2)	C10—C11—C12—C13	0.4 (3)
N1—C4—C5—C6	−179.52 (12)	C9—C8—C13—C12	0.1 (2)
C2—C1—C6—C5	−1.57 (19)	O6—C8—C13—C12	174.88 (14)
C2—C1—C6—N3	178.72 (12)	C11—C12—C13—C8	−0.5 (2)

### (II) Phenyl N-(3,5-dinitrophenyl)carbamate Hydrogen-bond geometry (Å, º)

**Table:**

*D*—H···*A*	*D*—H	H···*A*	*D*···*A*	*D*—H···*A*
N3—H3*A*···O5^i^	0.86	2.07	2.8836 (15)	157

Symmetry code: (i) −*x*+1, *y*−1/2, −*z*+1/2.
